# Migration in Namibia and its association with HIV acquisition and treatment outcomes

**DOI:** 10.1371/journal.pone.0256865

**Published:** 2021-09-02

**Authors:** Andrea Low, Karam Sachathep, George Rutherford, Anne-Marie Nitschke, Adam Wolkon, Karen Banda, Leigh Ann Miller, Chelsea Solmo, Keisha Jackson, Hetal Patel, Stephen McCracken, Sally Findley, Nicholus Mutenda

**Affiliations:** 1 ICAP at Columbia University, Mailman School of Public Health, Columbia University, New York, NY, United States of America; 2 Institute for Global Health Sciences, University of California, San Francisco, CA, United States of America; 3 Ministry of Health and Social Services, Windhoek, Namibia; 4 Centers for Disease Control and Prevention, Windhoek, Namibia; 5 Division of Global HIV and Tuberculosis, Centers for Disease Control and Prevention, Center for Global Health, Atlanta, GA, United States of America; International AIDS Vaccine Initiative, UNITED STATES

## Abstract

**Background:**

In the 21^st^ century, understanding how population migration impacts human health is critical. Namibia has high migration rates and HIV prevalence, but little is known about how these intersect. We examined the association between migration and HIV-related outcomes using data from the 2017 Namibia Population-based HIV Impact Assessment (NAMPHIA).

**Methods and findings:**

The NAMPHIA survey selected a nationally representative sample of adults in 2017. All adults aged 15–64 years were invited to complete an interview and home-based HIV test. Recent infection (<130 days) was measured using HIV-1 LAg avidity combined with viral load (>1000 copies/mL) and antiretroviral analyte data. Awareness of HIV status and antiretroviral use were based on self-report and/or detectable antiretrovirals in blood. Viremia was defined as having a viral load ≥1000 copies/mL, including all participants in the denominator regardless of serostatus. We generated community viremia values as a weighted proportion at the EA level, excluding those classified as recently infected. Significant migrants were those who had lived outside their current region or away from home >one month in the past three years. Recent cross-community in-migrants were those who had moved to the community <two years ago. Separate analyses were done to compare significant migrants to non-migrants and recent cross-community in-migrants to those who in-migrated >two years ago to determine the association of migration and timing with recent infection or viral load suppression (VLS). All proportions are weighted. Of eligible adults, we had HIV results and migration data on 9,625 (83.9%) of 11,474 women and 7,291 (73.0%) of 9,990 men. Most respondents (62.5%) reported significant migration. Of cross-community in-migrants, 15.3% were recent. HIV prevalence was 12.6% and did not differ by migration status. Population VLS was 77.4%. Recent cross-community in-migration was associated with recent HIV infection (aOR: 4.01, 95% CI 0.99–16.22) after adjusting for community viremia. Significant migration (aOR 0.73, 95% CI: 0.55–0.97) and recent cross-community in-migration (aOR 0.57, 95% CI: 0.35–0.92) were associated with lower VLS, primarily due to lack of awareness of HIV infection. The study was limited by lack of precise data on trajectory of migration.

**Conclusions:**

Despite a high population-level VLS, Namibia still has migrant populations that are not accessing effective treatment for HIV. Targeting migrants with effective prevention and testing programs in communities with viremia could enable further epidemic control.

## Introduction

The southern African nation of Namibia has both high migration rates and a large burden of HIV, yet little is known about how these factors intersect. The population is highly dispersed, and urbanization, poverty, and climate events, such as flooding or drought, require frequent adaptation including migration. Migration is a key driver of HIV transmission in sub-Saharan Africa, in part due to social disruption leading to high-risk behaviors such as sexual partner concurrency and transactional sex [1–4]. Migration also has been associated with delayed HIV diagnosis, and poor linkage and retention in care, with the potential to reverse gains in epidemic control associated with improved antiretroviral therapy (ART) coverage [5,6]. However, in some settings, labor migration is associated with improved incomes and better access to health services, particularly when movement is to urban areas [7–9]. Furthermore, patterns of migration have been shown to impact sexual networks, which contribute to risk of infection and transmission corridors [10,11]. Increased HIV risk has also been associated with longer distances traveled or time away from home [4,12]. Vulnerability associated with migration is therefore highly contextual [10,12–14]. Understanding the diversity of patterns of migration and how it impacts HIV-related behaviors and treatment access is critical to optimizing the HIV response.

In 2015, the government of Namibia launched an acceleration plan designed to achieve the UNAIDS 90-90-90 targets (90% of HIV-positive individuals know their status; of these, 90% are receiving ART; and of these, 90% [i.e., 73% of all HIV-positive individuals] have viral load suppression [VLS]) through decentralizing HIV care into community-based ART services and widespread viral load monitoring [15]. The 2017 Namibia Population-based HIV Impact Assessment (NAMPHIA) was conducted to assess progress toward the 90-90-90 goals and demonstrated that 77.4% of people living with HIV in Namibia had VLS (defined as HIV RNA<1,000 copies/mL) [16]. Namibia is thus one of the earliest countries worldwide to have exceeded the above target of 73% VLS. However, despite this achievement, there are still populations within Namibia lagging behind in terms of VLS, particularly men and adolescents[15,16]. There is also wide geographic variation, where certain regions have much lower levels of VLS but some communities within remain infectious, potentially acting as sources of new infections in other areas [10,12,16]. To achieve the new 95-95-95 goals and target further prevention and treatment initiatives efficiently, understanding the remaining hard-to-reach populations is imperative.

NAMPHIA is the first national survey in Namibia to collect data on migration and recent HIV infection and community infectiousness. To understand the patterns of HIV infection and the continuum of care, we used NAMPHIA data to describe sex-specific patterns of significant migration as well cross-community in-migration and to assess their associations with risk behaviors, HIV acquisition, and treatment.

## Materials and methods

### Survey design and population

The NAMPHIA survey employed a cross-sectional, two-stage, sampling design to obtain a nationally representative sample of adults aged 15–64 years and children aged 0–14 years living in households in Namibia in 2017. The sampling frame included all households in the country, based on the 2011 Population and Housing Census. In the second stage of sampling, households were randomly selected from each EA using an equal probability approach that allowed variation in the number of households depending on the size of the EA (Further details on sample design are provided in Supplemental Digital Content (SDC) 1 and in the NAMPHIA final report [16]). Consenting heads of household completed a household questionnaire, which included a roster of all residents and guests. People in selected households were eligible if they had slept in the house the night before. We asked eligible individuals to consent to a questionnaire that included sociodemographic and behavioral risk questions and to home-based HIV testing, and we documented informed consent at each stage via electronic signature. Parents/guardians provided consent for testing their children aged 0–9, and provided permission for approaching children aged 10–17, who were then asked for assent for an interview and HIV testing. The NAMPHIA protocol and data collection tools were approved by the Institutional Review Boards at the Namibia Ministry of Health and Social Services, Columbia University, University of California San Francisco, and the US Centers for Disease Control and Prevention (CDC).

### Procedures

Survey staff administered the questionnaire during face-to-face interviews using Google Nexus-9 tablets. The core and optional PHIA questionnaire modules were derived from prior widely used surveys in sub-Saharan Africa, including the Demographic and Health surveys. The NAMPHIA questionnaire was subjected to interal cognitive review with local staff and then reviewed for consistency and ease of understanding by field staff during training. The adult questionnaire included questions on lifetime and recent sexual behaviors and characteristics of the three most recent sexual partners from the past 12 months. To assess the impact of different types of migration, we used two indices: 1) to capture significant movement in terms of distance or duration, we defined significant migrants as those who reported ever having lived outside Namibia or in another region inside Namibia, or who reported living away from home continuously for more than 1 month in the past 3 years, and 2) to capture cross-community migration inflows and the impact of timing, we defined in-migrants as those who reported coming from another community during the survey, with recent cross-community in-migrants defined as those who reported living in their current community for <2 years [2,17], and long-term cross-community in-migrants defined as those who reported having moved there ≥2 years ago but were not born there (see SDC 2 for construction of variables). In-migrants did not specify their prior place of residence and therefore could include those without significant mobility. Alcohol use was assessed using the abbreviated version of the Alcohol Use Disorders Identification Test (AUDIT-C), with use classified as non-drinking, moderate, or hazardous, the latter including binge drinking (≥six drinks on one occasion at least monthly) [18].

Survey staff conducted HIV testing using Determine HIV-1/2 Rapid Test (Alere, Waltham, MA, USA), confirmed with the Uni-Gold HIV Test (Trinity Biotech, Bray, Ireland). Discordant results were retested with Clearview Complete (Inverness Medical, Waltham, MA, USA). All HIV-positive results were verified using the Geenius HIV 1/2 supplemental assay (Bio-Rad, Hercules, CA, USA). We quantified HIV-1 RNA in plasma and dried blood spots in HIV-seropositive samples using real-time polymerase chain reaction (Cobas Taqman, Roche, Indianapolis, IN, USA), with VLS defined as <1000 copies/mL. We tested HIV-positive dried blood spots for antiretroviral analytes for the three most commonly prescribed antiretrovirals with long half-lives (lopinavir, efavirenz, and nevirapine) using high-resolution liquid chromatography at the University of Cape Town, South Africa. Recent HIV infection was diagnosed using the HIV-1 Limited Antigen Avidity Enzyme Immunoassay (Sedia Biosciences, Portland, OR, USA) on HIV-positive specimens. Samples with a normalized optical density <1.5 that were not virally suppressed and did not have detectable antiretroviral analytes were classified as recent infection, with a mean duration of infection of 130 days (95% confidence interval [CI]: 118–142) [16].

### Statistical analysis

We calculated weights based on sampling design, including probabilities of household and individual selection, adjusted for non-response at the household, individual and biomarker levels per previously described methods [19]. We performed statistical analyses in Stata version 15.1 (College Station, TX) using weighted data, with jackknife replicate weights for variance estimation. The analyses were restricted to adults with HIV serostatus results. Poverty was defined as being in the lower two wealth quintiles. The UNAIDS 90-90-90 indicators were based on self-report and detection of antiretrovirals; those who tested positive for antiretrovirals were classified as aware and receiving ART regardless of self-reported status [16]. We assessed associations using logistic regression according to a proximate determinants framework where our two different indices of migration were examined as correlates of different outcomes, namely HIV risk behaviors, the treatment cascade, and VLS and recent HIV infection. As the literature indicates that community viremia, defined as the proportion of the population who have a viral load ≥1000 copies/mL and are therefore infectious [20–22], is one of the strongest predictors of new HIV infections in that community [23,24], we included it in our models examining the correlates of recent HIV infection. We generated community viremia values as a weighted proportion of participants at the EA level with viremia, excluding those classified as recently infected to avoid introducing bias. Significant migrants were compared to those without any reported substantial migration (classified as non-migrants). To assess the timing of risk of cross-community in-migration, we compared recent cross-community in-migrants to longer-term cross-community in-migrants. The analysis of our migration indices and their associations with risk behaviors was stratified by sex, based on literature suggesting that the impact of migration on behavior is very gendered.[1,2,4] Our analysis of the associations between our migration indices and the HIV-specific outcomes revealed that the associations did not vary by sex, and we therefore pooled the data; however we adjusted the models for sex as well as age as a continuous variable. Multivariable models were built for each index of migration as an exposure, examining their associations with VLS and recent infection as dependent variables. These models were builtincluding all variables with a p<0.10 on univariable analysis. All presented data are weighted proportions and crude numbers, aside from unweighted response rates.

### Construction of maps

HIV prevalence and community viremia maps were generated by inverse density weighting using georeferenced weighted prevalence of HIV and viremia data aggregated at the level of the enumeration area (EA), with all cases linked to the centroid of the selected EA, in ArcGIS 10.2 (Redlands, CA, USA).[25] Internal migration flow data derived from census micro-data were downloaded from the WorldPop project (Southampton, UK) [26,27].

## Results

Of 12,689 selected households, 10,921 (86.1%) were occupied, and 9,315 (85.3%) completed a household interview. Of eligible adults, we had HIV results and migration data on 9,625 (83.9%) of 11,474 women and 7,291 (73.0%) of 9,990 men. The median age of participants was 30 years (interquartile range [IQR], 22–41 years), and 42.3% resided in rural areas. Overall, 6.1% reported having living outside Namibia, 52.5% had lived in another region, and 28.8% had lived away from home for more than a month in the past three years, for a total of 62.5% of adults classified as significant migrants (Table 1). Of the 10,325 cross-community in-migrants, 15.3% were recent; there was no difference by sex. Both male and female significant migrants were younger, more urban, educated, and employed and were less likely to be poor than non-migrants, whereas recent cross-community in-migrants were younger than long-term cross-community in-migrants, but their educational differences were not as pronounced as among significant migrants vs. non-migrants. Female recent cross-community in-migrants were wealthier than their long-term cross-community in-migrant counterparts, a difference not found among men. In both sexes, significant migrants were less likely to be married than non-migrants, and had higher numbers of lifetime sexual partners. Recent cross-community in-migrants were also less likely to be married than long-term cross-community in-migrants, but there was no difference in number of partners. There were variations in migration patterns by occupation (SDC3 provides details on behaviors by sex and occupation), where inter-regional migration was most common in women working in manufacturing (67.8%) and in men employed in fishing (92.0%).

**Table 1:** Characteristics of adults aged 15–64 years who participated in the 2017 Namibia opulation-based HIV Impact Assessment (NAMPHIA).

	Women					Men				
	Total^a^ (n = 9,625)	Non- migrants (n = 4,655)	Significant migrants (n = 4,970)^b^	Recent CC in-migrants (n = 818)^c^	Longer CC in-migrants (n = 5,021)	Total^a^ (n = 7,291)	Non-migrants (n = 2,810)	Significant migrants (n = 4,481)^b^	Recent CC in-migrants (n = 704)^c^	Longer CC in-migrants (n = 3,782)
**Age (years)**										
Median age (IQR)	30 (22–42)	32 (21–45)	29 (22–40)	25 (21–32)	31 (23–43)	29 (22–41)	27 (19–41)	30 (23–40)	26 (21–33)	31 (23–42)
15–24	3,012 (33.5%)	1,456 (33.8%)	1,556 (33.2%)	373 (47.0%)	1,433 (31.1%)	2,546 (34.8%)	1,202 (44.4%)	1,344 (30.2%)	279 (40.4%)	1,217 (31.4%)
25–34	2,511 (27.3%)	973 (21.7%)	1,538 (31.4%)	285 (34.3%)	1,275 (26.9%)	1,791 (28.1%)	505 (19.6%)	1,286 (32.2%)	242 (36.3%)	899 (27.3%)
35–44	1,875 (18.7%)	914 (18.6%)	962 (18.8%)	98 (11.7%)	1,026 (19.7%)	1,440 (18.9%)	481 (16.6%)	959 (20.0%)	95 (12.5%)	802 (21.0%)
45–54	1,297 (12.6%)	732 (15.1%)	565 (10.8%)	45 (5.1%)	752 (13.8%)	931 (11.7%)	351 (11.1%)	580 (12.0%)	54 (6.6%)	537 (13.4%)
55–64	929 (7.9%)	580 (10.8%)	349 (5.8%)	17 (1.8%)	535 (8.6%)	583 (6.6%)	271 (8.4%)	312 (5.7%)	34 (4.3%)	327 (6.8%)
			p<0.0001		p<0.0001			p<0.0001		p<0.0001
**Residence**										
Urban	4,034 (57.7%)	1,581 (45.8%)	2,453 (66.5%)	387 (64.2%)	2,082 (58.3%)	2,906 (57.7%)	847 (42.4%)	2,059 (65.0%)	277 (59.7%)	1,537 (59.7%)
Rural	5,591 (42.3%)	3,074 (54.2%)	2,517 (33.5%)	431 (35.8%)	2,939 (41.7%)	4,385 (42.3%)	1,963 (57.6%)	2,422 (35.1%)	427 (40.3%)	2,245 (40.4%)
			p<0.0001		p = 0.0115			p<0.0001		p = 0.9784
**HH poverty** ^**d**^										
Not poor	4,711 (60.7%)	1,800 (47.9%)	2,911 (70.1%)	505 (72.3%)	2,471 (61.1%)	3,538 (60.6%)	1,047 (45.8%)	2,491 (67.6%)	329 (59.0%)	1,871 (62.2%)
Poor	4,914 (39.3%)	2,855 (52.1%)	2,059 (29.9%)	313 (27.7%)	2,550 (38.9%)	3,752 (39.4%)	1,763 (54.2%)	1,990 (32.4%)	375 (41.0%)	1,911 (37.8%)
			p<0.0001		p = 0.0001			p<0.0001		p = 0.2939
**Receipt of economic support in past 3 months**										
Yes	3,780 (34.2%)	1,947 (38.3%)	1,833 (31.2%)	238 (23.4%)	2,018 (35.3%)	2,525 (31.1%)	1,142 (39.8%)	1,383 (27.0%)	160 (19.1%)	1,317 (30.7%)
No	5,845 (65.8%)	2,708 (61.7%)	3,137 (68.8%)	580 (76.6%)	3,003 (64.7%)	4,766 (68.9%)	1,668 (60.3%)	3,098 (73.0%)	544 (80.9%)	2,465 (69.3%)
			p<0.0001		p<0.0001			p<0.0001		p<0.0001
**Educational status** ^**e**^										
None	813 (6.1%)	543 (9.2%)	271 (3.8%)	74 (5.6%)	396 (5.7%)	820 (8.2%)	404 (11.2%)	416 (6.8%)	119 (12.4%)	429 (8.2%)
Primary	2,718 (22.7%)	1,629 (30.6%)	1,089 (17.0%)	189 (18.4%)	1,476 (23.5%)	2,360 (25.4%)	1,088 (33.7%)	1,272 (21.5%)	222 (23.4%)	1,245 (25.9%)
Secondary	5,383 (59.3%)	2,314 (54.5%)	3,069 (62.8%)	474 (61.7%)	2,733 (57.4%)	3,606 (54.7%)	1,234 (51.0%)	2,372 (56.5%)	303 (50.1%)	1,821 (53.2%)
Post-secondary	685 (11.9%)	149 (5.8%)	536 (16.4%)	78 (14.4%)	399 (13.4%)	486 (11.7%)	74 (4.2%)	412 (15.3%)	58 (14.2%)	274 (12.6%)
			p<0.0001		p = 0.0346			p<0.0001		p = 0.0387
**Formal Employment**										
Not employed	6,472 (62.9%)	3,532 (72.9%)	2,940 (55.5%)	493 (58.0%)	3,319 (61.5%)	3,537 (46.2%)	1,775 (62.8%)	1,762 (38.3%)	244 (40.0%)	1,799 (43.3%)
In past 12mos, not currently	1,030 (11.4%)	348 (8.4%)	682 (13.7%)	106 (14.4%)	571 (12.1%)	1,180 (16.8%)	299 (11.1%)	881 (19.6%)	118 (16.2%)	657 (18.7%)
Currently employed	2,110 (25.7%)	762 (18.7%)	1,348 (30.8%)	217 (27.6%)	1,122 (26.4%)	2,563 (37.0%)	727 (26.1%)	1,836 (42.1%)	341 (43.7%)	1,322 (38.0%)
			p<0.0001		p = 0.2088			p<0.0001		p = 0.1118
**Marital status**										
Never married	5,161 (58.0%)	2,410 (56.2%)	2,751 (59.4%)	490 (65.8%)	2,626 (57.4%)	4,171 (60.0%)	1,741 (65.9%)	2,430 (57.2%)	432 (66.8%)	2,095 (57.4%)
Married	3,358 (32.3%)	1,687 (33.5%)	1,671 (31.4%)	270 (27.7%)	1,771 (32.5%)	2,569 (33.4%)	881 (29.0%)	1,688 (35.4%)	197 (25.4%)	1,407 (36.1%)
Divorced/widowed	1,019 (9.7%)	513 (10.3%)	506 (9.3%)	55 (6.5%)	557 (10.1%)	491 (6.6%)	161 (5.1%)	330 (7.3%)	63 (7.8%)	245 (6.6%)
			p = 0.0496		p = 0.0014			p<0.0001		p = 0.0001
**Number of lifetime sexual partners**										
Mean (95% CI)	2.7 (2.5–2.9)	2.4 (2.3–2.5)	3.0 (2.7–3.3)	3.4 (2.5–4.3)	2.6 (2.4–2.7)	7.5 (7.0–8.1)	5.0 (4.4–5.6)	8.8 (8.0–9.5)	7.9 (6.6–9.2)	7.8 (7.0–8.6)
			p = 0.002		p = 0.059			p<0.001		p = 0.926
**Male circumcision**										
Yes	**~**	**~**	**~**	**~**	**~**	2,687 (39.3%)	934 (36.1%)	1,753 (40.8%)	290 (39.2%)	1,360 (39.4%)
No						4,478 (60.7%)	1,805 (63.9%)	2,673 (59.2%)	408 (60.8%)	2,363 (60.6%)
								p = 0.0063		p = 0.9534
**HIV prevalence**										
Positive	1,689 (15.7%)	844 (16.9%)	845 (14.7%)	123 (15.2%)	886 (14.0%)	756 (9.3%)	307 (10.6%)	449 (8.7%)	45 (5.4%)	412 (9.7%)
			p = 0.0257		p = 0.3821			p = 0.0354		p = 0.0057

Data are presented as medians (IQR) or numbers. All percentages are weighted. Rows may not equal total due to missing data. P-values for comparisons between signifcant and non-migrants, and between recent and longer-term cross-community in-migrants, were estimated using Chi-square test for comparisons of categorical values for and Student’s t-test for comparison of means.

^a^ Total includes adults aged 15–64 with an HIV test result and data on ever migration.

^b^ Significant migrant is defined as anyone who has lived outside the country, in another region, or away from home for at least 1 month in the past 3 years.

^c^ Recent cross-community in-migrant is defined as anyone who has moved to their current community or town in the past 2 years.

^d^Poverty is defined as the poorest two wealth quintiles.

^e^ Educational attainment reflects highest level attended.

IQR, interquartile range; HH, household; CI, confidence interval; CCcross-community.

Note that cross-community in-migration might include intra-regional migration and therefore does not exclude those classified as non-migrants in our other migration index.

**Fig 1:**
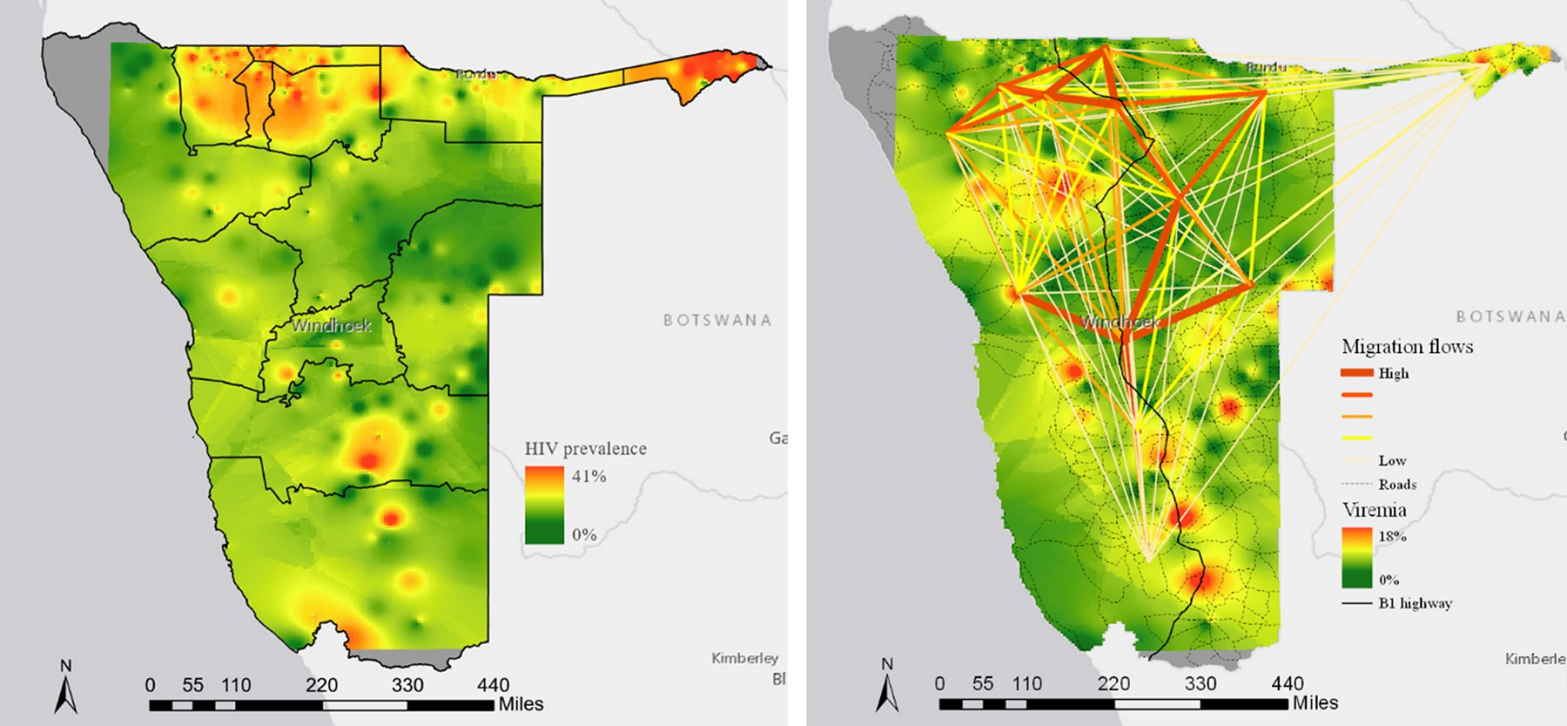
Distribution of HIV infection, and viremia across Namibia, 2017. a) Prevalence of HIV in Namibia, 2017. Maps with spatial interpolation to estimate prevalence of each outcome in adults aged 15–64 years using inverse density weighting (Namibia, 2017). (a) Weighted HIV prevalence (%) in adults. b) Prevalence of HIV viremia with migration flows overlaid. Weighted prevalence of HIV viremia in all adults (i.e., proportion of the population with an HIV viral load >1000 copies/mLml, regardless of serostatus). The migration flows were derived from Worldpop micro-census data. The black line indicates the B1 highway from South Africa to Angola.

Migration patterns varied considerably by region, with the highest proportion reporting having previously lived in coastal Erongo, northern Omusati or Ohangwena, or in urban Khomas (Table SDC 4 in S1 File provides a regional breakdown). The highest prevalence of international migrants was in Zambezi (19.8%,). VLS ranged from 55.2% in Kunene to 86.2% in Ohangwena (SDC 4); at the EA level, HIV prevalence ranged from 0% to 45.7% (mean, 12.3%) and viremia ranged from 0% to 29.1% (mean, 3.1%), with slightly lower interpolated aggregate values (Fig 1A and 1B). HIV prevalence was highest along the northern border with Angola and in Zambezi (Fig 1A), whereas viremia was most common along the B1 highway from South Africa to Angola (Fig 1B).

Compared to non-migrants, both female and male significant migrants had higher odds of multiple sexual partners in the past yearc, but among the in-migrants only female recent cross-community in-migrants were more likely to have multiple recent sexual partners (Table 2). Significant migrants were more likely to have had multiple lifetime sexual partners than the non-migrants, a pattern also found among recent cross-community in-migrants. Significant migration was also associated with participating in commercial sex, both in women selling sex (Odds Ratio [OR] 3.00, 95% CI 1.34–6.71) and men buying it (OR 1.65, 95% CI 1.18–2.31). Although there were trends for increased hazardous drinking in all migrants, this trend was significant only in male significant migrants (OR 1.42, 95% CI 1.22–1.65). More significant migrants having been tested in the past 12 months than non-migrants, and female recent in-migrants were more likely to report testing in the past year than male recent cross-community in-migrants. HIV prevalence varied significantly by migration status only for male significant migrants, who had lower prevalence than non-migrants (OR 0.78, 95% CI 0.64–0.95).

**Table 2:** Associations between the two indices of migration and HIV risk behaviors by sex among participants in the 2017 Namibia Population-based HIV Impact Assessment (NAMPHIA).

	Age-adjusted OR (95% CI)			
	Female		Male	
Outcome	Significant migrant vs non-migrant^a^ (n = 9,621)	Recent vs longer term CC in-migrant^b^ (n = 5,839)	Significant migrant vs non-migrant^a^ (n = 7,290)	Recent vs longer term CC in-migrant^b^ (n = 4,486)
**Educational attainment**				
None/Primary	**1.0**	1.0	**1.0**	1.0
Secondary or more^c^	**2.41 (2.11–2.76)** ***	1.00 (0.81–1.24)	**2.18 (1.89–2.52)** ***	0.85 (0.67–1.08)
**Recent Employment Status**				
No formal work	**1.0**	**1.0**	**1.0**	**1.0**
Work in the past year	**2.30 (2.04–2.59)** ***	**1.32 (1.09–1.60)** **	**2.70 (2.39–3.06)** ***	**1.34 (1.05–1.72)** *
**Number of sexual partners in the past year**				
None/One	**1.0**	**1.0**	**1.0**	1.0
More than one	**1.60 (1.18–2.18)** **	1.59 (1.00–2.52) *	**2.54 (2.06–3.12)** ***	1.12 (0.88–1.43)
**Number of lifetime sexual partners**				
None/One	**1.0**	**1.0**	**1.0**	**1.0**
More than one	**1.80 (1.58–2.04)** ***	**1.61 (1.32–1.96)** ***	**3.06 (2.57–3.64)** ***	**2.09 (1.58–2.78)** ***
**History of commercial sex**				
No	**1.0**	1.0	**1.0**	1.0
Has bought or sold sex	**3.00 (1.34–6.71)** **	0.70 (0.11–4.48)	**1.65 (1.18–2.31)** **	1.57 (0.88–2.79)
**Alcohol use**				
None/moderate drinking	1.0	1.0	**1.0**	1.0
Hazardous drinking^**d**^	1.13 (0.95–1.34)	1.31 (0.99–1.73)	**1.42 (1.22–1.65)** ***	1.30 (0.99–1.69)
**HIV testing in past 12 months**				
No	**1.0**	**1.0**	**1.0**	1.0
Yes	**1.43 (1.27–1.60)** ***	**1.26 (1.03–1.54)** *	**1.57 (1.37–1.81** )***	0.96 (0.77–1.20)
**Biological HIV outcomes**				
**Recent HIV infection**				
HIV negative	1.0	1.0	1.0	1.0
Recent infection	1.16 (0.31–4.37)	3.01 (0.40–22.50)	0.83 (0.14–4.87)	4.38 (0.76–25.26)
**Prevalent HIV infection**				
HIV negative	1.0	1.0	**1.0**	1.0
HIV positive^e^	0.94 (0.80–1.10)	1.25 (0.97–1.59)	**0.78 (0.64–0.95)** *	0.67 (0.42–1.06)

* p≤0.05

** p≤0.01

***p≤0.001. Results in bold indicate a statistically significant result.

Data were survey weighted using jackknife replicate weights to estimate variance. All models were constructed using logistic regression of weighted data, adjusted for age as a continuous variable.

aReference is anyone without a significant migration history.

^b^Reference is anyone who moved to the community over 2 years ago^.^

^c^Education is based on highest level attended.

^d^Defined using the AUDIT-C scale.

^e^ Excluding those defined as recently infected.

CI, confidence interval; OR, odds ratio.

There was substantial heterogeneity in HIV infection by occupation (Tables SDC3a/b in S1 File), with the highest HIV prevalence in domestic workers among women (19.8%, 95% CI 15.8%–23.8%) and in fishermen among men (13.8%, 95% CI 6.0%–21.7%).

Despite reporting more recent HIV testing, HIV-positive significant migrants had significantly lower awareness (83.3%) than non-migrants (89.6%, p = 0.002; SDC5 provides a 90-90-90 bar chart by migrant type), with the lowest rates among recent (70.9%) compared to long-term cross-community in-migrants (87.7%, p = 0.0003). Compared to non-migrants, there were significantly lower odds of detection of antiretrovirals in significant migrants (OR 0.67, 95% CI 0.51–0.88; Table 3), and significant migrants had almost 40% lower odds of VLS (OR 0.67, 95% CI 0.50–0.88). Recent cross-community in-migrants also had lower awareness (OR 0.45, 95% CI 0.26–0.79) and lower odds of VLS (OR 0.54, 95% CI 0.34–0.86) than long-term cross-community in-migrants.

**Table 3:** Associations between the two indices of migration and the HIV-treatment cascade among people living with HIV in the 2017 Namibia Population-based HIV Impact Assessment (NAMPHIA).

	Adjusted odds ratio (95% CI)	
Outcome	Significant vs non- migrants^a^ (n = 2,440)	Recent vs longer term in-migrants^b^ (n = 1,462)
**Awareness of sero-status**		
**Unaware**	**1.0**	**1.0**
**Aware**	**0.62 (0.44–0.88)** **	**0.45 (0.26–0.79)** **
**Antiretroviral status**		
**None**	**1.0**	**1.0**
**On Antiretrovirals**	**0.65 (0.48–0.87)** **	**0.48 (0.28–0.80)** **
**Detectable antiretrovirals**		
**None detected**	**1.0**	**1.0**
**ARVs detected**	**0.67 (0.51–0.88)** **	**0.48 (0.31–0.75)** **
**Detectable antiretrovirals in those aware**		
**None detected**	1.0	1.0
**ARVs detected**	0.75 (0.47–1.18)	0.68 (0.37–1.23)
**Viral load <1000 copies/ml (VLS)**		
**No**	**1.0**	**1.0**
**Yes**	**0.67 (0.50–0.88)** **	**0.54 (0.34–0.86)** **
**VLS in those on ART**		
**No**	1.0	1.0
**Yes**	0.84 (0.58–1.22)	0.67 (0.35–1.28)

Data were survey weighted using jackknife replicate weights to estimate variance. All models were constructed using logistic regression of weighted data, adjusted for age as a continuous linear variable, and sex.

^a^Reference is anyone with no reported migration history.

^b^Reference is anyone who moved to the community over 2 years ago.

* p≤0.05

** p≤0.01

***p≤0.001. Results in bold indicate a statistically significant result.

Awareness of HIV-positive sero-status was based on self-report and/or having detectable antiretrovirals, and ART status was based on self-report and/or having detectable antiretrovirals.

CI, confidence interval; ART, antiretroviral therapy.

On multivariable analysis, the reduced likelihood of having VLS among migrants persisted after controlling for other sociodemographic influences on VLS. In both models, significant migrants (adjusted OR [aOR] 0.73, 95% CI 0.55–0.97; Table 4) or recent cross-community in-migrants (aOR 0.57, 95% CI 0.35–0.92) had lower odds of VLS, as well as those who had worked in the past 12 months. In both analyses, women and older people were more likely to be virally suppressed, and hazardous drinking was the strongest predictor of lack of VLS. The inclusion of urban/rural residence in the model did not affect these results.

**Table 4:** Multivariable models of factors predicting recent HIV infection and viral load suppression by migration pattern in the 2017 Namibia Population-based HIV Impact Assessment (NAMPHIA).

Predictors of VLS and recent HIV infection by Migration Type					
Significant migrant vs. Non-migrant			Longer term vs. recent in-migrant		
	VLS aOR (95% CI) (n = 2,433)	Recent HIV infection aOR (95%CI) (n = 9,820)^a^		VLS aOR (95% CI) (n = 1,461)	Recent HIV infection aOR (95%CI) (n = 5,845)^a^
**Non—migrant**	**1.0**	1.0	**Longer-term in-migrant**	**1.0**	**1.0**
**Significant migrant**	**0.73 (0.55–0.97)** *	1.11 (0.30–3.50)	**Recent in-migrant**	**0.57 (0.35–0.92)** *	**4.01 (0.99–16.22)** *
**Age (per year increase)**	**1.05 (1.04–1.06)** ***	0.98 (0.95–1.02)	**Age (per year increase)**	**1.06 (1.04–1.08)** ***	1.01 (0.97–1.05)
**Male**	**1.0**	**1.0**	**Male**	**1.0**	1.0
**Female**	**1.92 (1.49–2.47)** ***	**4.55(1.45–14.25)** **	**Female**	**2.23 (1.54–3.22)** ***	2.26 (0.64–7.98)
**Wealth quintile (per quintile increase)**			**Wealth quintile (per quintile increase)**		**0.48 (0.29–0.82)** **
**No work in past year**	**1.0**		**No work in past year**	**1.0**	
**Worked in past year**	0.70 (0.54–0.90)**		**Worked in past year**	**0.64 (0.45–0.92)** **	
**No or moderate drinking**	**1.0**		**No or moderate drinking**	**1.0**	
**Hazardous drinking**	**0.33 (0.22–0.49)** ***		**Hazardous drinking**	0.29 (0.17–0.48)*	
**Community viremia** ^**a**^ **(per 1% increase)**			**Community viremia** ^**a**^ **(per 1% increase)**		1.11 (0.99–1.23)

* p< = 0.05

** p≤0.01

***p<0.001. Results in bold indicate a statistically significant result.

Data were survey weighted using jackknife replicate weights to estimate variance. Multivariable models were constructed using logistic regression of weighted data, excluding any variables with a p-value >0.10 on univariable analysis. These variables include urban vs rural residence and educational attainment.

^a^ Community viremia was calculated as a weighted proportion of those who were viremic, defined as HIV viral load >1000 copies/ml, in all those not classified as newly infected, generated at the EA level.

VLS, viral load suppression; aOR, adjusted odds ratio; CI, confidence interval; HH, household; NI, not included.

In the multivariable model adjusted for community viremia, recent in-migration was associated with a four-fold increase in odds of recent infection (aOR 4_._01, 95% CI 0_._99–16_._22, Table 4). Increasing wealth was protective against recent infection (aOR 0.48, 95% CI 0_._29–0_._82). In the model with SM, only female sex increased the odds of recent infection (aOR 4.66, 95% CI 1_._51–14_._42).

## Discussion

To our knowledge, our study is the first to evaluate the associations between migration, HIV acquisition and VLS in Namibia, one of the first countries globally to achieve the 90-90-90 goals [15,16]. Population-level contextual factors and individual data allowed us to provide new understanding of the impact of migration on HIV risk. The nationally-representative sample ensured that we are capturing a cross-section of migrants at home or a secondary residence, ensuring that we can evaluate whether they have truly defaulted on treatment or have sought care elsewhere, which is a critical gap in other studies [28]. Despite high rates of VLS overall, we found that health vulnerabilities still affect migrants, as evidenced by lower VLS in both types of migrants and increased recent HIV infection in those recently moving into a community. Unlike studies in other countries, we did not find any association between HIV prevalence and migration [4,29,30], likely reflective of the heterogeneity of migration patterns and their associated impact on socioeconomic status and subsequent adoption of risk behaviors, as well as our inability to sort out temporal sequences between migration and HIV status changes for all but the most recent infections. Despite this, we demonstrated that, after adjusting for community viremia, recent in-flow migration was associated with four times higher odds of recent infection.

Migration has long been identified as a risk for HIV infection due to family separation and loss of social support, leading to risky behaviors, such as substance abuse, sexual concurrency, and transactional sex, compounded by lack of access to HIV prevention, testing, and care [31–33]. Despite the fact that significant migrants were more educated, and more likely to be employed, they were more likely to have more sexual partners and to engage in commercial sex work, risky behaviors which tend to be associated with income loss, particularly in women [34]. The behavioral changes were similar in recent in-migrants, although the beneficial socioeconomic factors were not; male recent in-migrants in particular were not more educated Our findings underscore the concern that migration is often associated with a search for new sexual partners.

HIV prevalence and viremia corresponded to the major B1 highway, which links South Africa to Angola. Seaports, like Namibia’s Walvis Bay, have also historically been identified as reservoirs of HIV infection because port communities act as a hub linking mobile communities (e.g., seafarers and transport workers) with the more settled Namibian population [35]. The transitory population in these seaports engage in risky behaviors, such as hiring sex workers and abusing alcohol [36]. Our data found that fishermen had the highest prevalence of viremia of all occupations, and transport workers also had high HIV prevalence, similar to findings from other studies in the region [37,38]. The higher HIV prevalence seen in Zambezi and the north likely reflects the historical role of trade and cross-border movement in HIV transmission [39]. It is noteworthy that these transient populations have been targeted by government programs since 2015 [39], but high levels of viremia persist, indicating that more innovative methods will be needed to properly address gaps.

Similar to other African studies [2,17], recent in-migration increased the odds of recent HIV infection; however, new infections were only found where the inflow was into a viremic community, supporting the findings of other studies that showed that population viral load is a key determinant in HIV infection [23], and that the role of migration in HIV risk is highly contextual [14]. Our finding that mobility was not associated with higher HIV prevalence overall could reflect the fact that traditional drivers of infection have been mitigated by high levels of viral control at the population level in Namibia[14]. There also might be some protective effect of higher education and income in significant migrants, offsetting behavioral risk, and, contrary to other settings [30], PLHIV in Namibia might be less likely to migrate to avoid disrupting treatment.

The lower odds of VLS among those reportingsignificant migration and recent in-migration is similar to that seen in other studies [5], but our study further shows that it was mainly driven by lack of awareness. The contrast with the higher odds of reporting recent testing in all female migrants and in male significant migrants may reflect social desirability bias associated with recent testing campaigns, rather than actual testing experience. Prevalence of VLS also was highly heterogeneous across occupations and could reflect workplaces providing HIV care in some settings, or that certain professions are more common in young people, who tend to have lower VLS rates overall [15]. Because few participants reported international migration, we were not able to compare external and internal migration, but this comparison warrants further investigation to identify barriers to access.

This study had several limitations. The cross-sectional design limits the ability to determing the timing of migration compared to HIV acquisition or how it generates barriers to care. Further, the questionnaire did not capture location or timing of the most recent month outside the home or the location of prior residence in the case of recent cross-community in-migrants, requiring the generation of two different migration indices to allow the comparison of NAMPHIA data to other studies [2,4]. Although adjusting for urban residence did not alter our results, a more complex questionnaire would have enabled analysis of the effect of urban-rural movement, as well as more dynamic mapping of community context at different time points, as has been done in studies using cell phone data [14]. As others have shown that HIV-positive people are often more likely to move into HIV hotspots [12], understanding which communities are more likely to seed others is critical, but cannot be done from our data [10]. Furthermore, it is difficult to capture people living in informal settlements or in hotels, and under-sampling of mobile populations could have introduced bias. However, we did include guests and temporary workers in our eligibility criteria, and we adapted our sampling protocol to capture people who were living in temporary structures away from selected households if we could verify their identity with community leaders.

### Conclusions

In conclusion, our study shows that despite a high population-level VLS, Namibia still has populations not effectively accessing treatment for HIV. Although we did not find an association between migration and higher HIV prevalence, we did find an association between migration and recent HIV infection and lack of VLS. This should be further assessed using targeted surveys of priority populations, such as fishermen, transport workers, and domestic workers, to identify treatment needs and individualized constraints. In the meantime, as viremia appeared to correspond with the B1 highway, continued scale-up of testing and treatment at sites along this route might be an efficient way to interrupt transmission and improve access to care.

## Supporting information

S1 File Supplemental digital content. (DOCX)
