# Supplementary material for: Migration in Namibia and its association with HIV acquisition and treatment outcomes
Source: PLoS One. 2021 Sep 2;16(9):e0256865. doi: 10.1371/journal.pone.0256865 (PMC8412347; doi:10.1371/journal.pone.0256865)
Supplement: S1 File — (DOCX) [file pone.0256865.s001.docx]

**Supplemental Digital Content 1. Details on survey design**

The NAMPHIA survey employed a cross-sectional, two-stage, cluster sampling design to obtain a nationally representative sample of adults aged 15-64 years and children aged 0-14 years living in households in Namibia. The sampling frame included all households in the country, based on the 2011 Population and Housing Census.[[1](#_ENREF_1),[2](#_ENREF_2)] The first-stage sampling units were 465 enumeration areas (EAs) selected with probabilities proportionate to the number of households in the EA, with allocation to the 14 regions designed to achieve 30% precision around a national estimate of incidence and 95% confidence intervals (CI) of ±0.10 for regional estimates of viral load suppression (VLS) in individuals aged 15-49 years.

We estimated the minimum number of adult blood draws using the CDC incidence calculator with a predicted annual national incidence for adults aged 15-49 of 0^.^81% (2014 UNAIDS conservative estimate [lower CI]), and a mean duration of recent infections of 130 days. We assumed HIV prevalence rates of 14^.^0% in adults aged 15-49 years, based on the 2013 Namibia Demographic and Health Survey.[[3](#_ENREF_3)] A VLS rate of 50% was assumed in each region.

There was an assumed intra-cluster correlation of 0^.^05 for prevalence and VLS rates; we estimated that 12,585 households would yield a sample of 23,424 adults aged 15-49 years old agreeing to a blood draw. The estimated numbers of households, individuals and blood draws included adjustments for household vacancy and non-response, number of individuals per household, individual non-response, and refusal of blood testing or specimen loss, based on data derived from the Namibia Demographic and Health Survey of 2013.[[3](#_ENREF_3)]

All households within the boundaries of the selected EAs were listed by trained staff in February-March 2017. In the second stage of sampling, households were randomly selected from each EA using an equal probability approach that allowed variation in the number of households depending on the size of the EA between the time of the census and the survey household listing. On average, 25 households were selected in each EA.

**Supplemental Digital Content 2. Data sources and construction of variables**

The migration module included the following questions:

1. Have you ever lived outside Namibia? [YES/NO]

2. In the last 3 years, have you been away from home for more than one month at a time? [YES/NO/DON’TKNOW/REFUSED]

3. In the last 3 years, how many times away from home for more than one month? [NUMBER OF TIMES]

4. Now I'm going to ask you about the most recent time you were away from home for more than one month.

What was the main reason you were away from home that most recent time? [WORK/SCHOOL/FAMILY OBLIGATIONS/MEDICAL CARE/TRAVEL/OTHER]

5. In what country/countries did you spend more than one month living in during the past year?

6. How long have you lived in this residence/ town? [WEEKS/MONTHS/YEARS/MY ENTIRE LIFE]

7. Have you ever lived in Namibia outside this region? [YES/NO/DON’T KNOW/REFUSED]

8. In which region/regions did you previously reside? [SELECT REGION FROM LIST]

9. In what industry do you work? [SELECT OCCUPATION FROM LIST]

10. When you were away, did you have access to any HIV treatment or care services? [YES/NO/DON’T KNOW/REFUSED]

A participant was classified as a significant migrant if they answered YES to any of questions 1,2, or 7.

The threshold for classification of recent cross-community in-migration was based on studies out of Tanzania and Uganda demonstrating that elevated HIV incidence is highest during the first two years after migration.[[4](#_ENREF_4),[5](#_ENREF_5)] A participant was classified as a recent cross-community in-migrant (RCC) if they answered that they had lived in this residence/town for less than 2 years, and a longer cross-community in-migrant (LCC) if they answered 2 years or more but not ‘my entire life’ to question 6.

The *socio-demographic characteristics* included residence, defined as urban vs rural, and wealth quintile, which was constructed using Principal Component Analysis (PCA) based on household assets and infrastructure, including the type of house construction, cooking fuel, toilet and water source. For variables at the individual-level, these included age and educational level, defined as the level attended, even if not completed. Employment status was based on reported recent status of paid work, where they were classified as currently enrolled in school, engaged in paid work in the past 12 months, both or neither. Marital status was defined as never married or having lived with a sexual partner, currently married or living with a partner, or no longer married, comprised of all who responded that they were currently separated, divorced or widowed.

*Behavioural variables* included asking whether participants had ever been tested for HIV and received the results, and if they had done so in the past 12 months, and female participants were asked about previous pregnancies and their outcomes. *Sexual behaviour variables* described the lifetime number of sexual partners, who could be partners with whom the participant engaged in either anal or vaginal sex acts. Among those who reported sexual activity in the past 12 months, the following characteristics were measured: how many partners, and for the three most recent partners, their relationship status with the participant (including casual partner, regular partner or husband). For condom use with an extramarital partner, the denominator was those who reported having an extramarital partner in the past 12 months.

**Supplemental Digital Content 3. Occupation, behaviors and HIV indicators by sex**

Previous studies have shown that different occupations and their communities are associated with substantial heterogeneity in HIV risk, driven in part by different mobility patterns and sexual behaviors [[6](#_ENREF_6),[7](#_ENREF_7)]. Mobile communities are very diverse, and the impact of mobility on HIV risk varies heavily by context, including how much time is spent in infection hot spots, legal status of migrants, and ability to access care while mobile, including through labor organizations [[7-11](#_ENREF_7)]. Coastal communities and sea-ports in Southern and Eastern Africa have been associated with concurrent partnerships and commercial sex work, and HIV transmission [[6](#_ENREF_6),[12](#_ENREF_12)]. We used reported occupational data to describe variation in HIV risk behaviors, HIV prevalence and infectiousness to allow comparison with other settings as well as to understand how this might impact our analysis of mobility and HIV transmission.

**Table SDC3a. Lifetime migration characteristics and HIV-related outcomes by occupation in women aged 15-64 years**

|  | **Proportion away from home for more than a month in the past 3 years % (95% CI)** | **Proportion who have lived in another region**  **% (95% CI)** | **Lifetime number of sex partners- mean (95% CI)** | **Proportion with a non-marital**  **partner in past year % (95% CI)** | **Proportion HIV-seropositive % (95% CI)** | **Proportion of PLHIV with VLS**  **% (95% CI)** | **Proportion of all participants with viremia**  **% (95% CI)** |
| --- | --- | --- | --- | --- | --- | --- | --- |
| **Occupation** |  |  |  |  |  |  |  |
| No occupation (n=6,558) | 24.3% (22.8-25.9%) | 41.5% (39.2-43.8%) | 2.42 (2.15-2.69) | **56.5% (54.2-58.7%)** | **17.0% (15.7-18.2%)** | 82.6% (79.8-85.4%) | 3.0% (2.4-3.5%) |
| Agriculture (n=260) | 28.4% (21.4-35.4%) | 52.0% (42.0-62.0%) | 3.69 (2.35-5.04) | 31.3% (21.8-40.9%) | **15.5% (10.3-20.7%)** | 86.7% (75.4-97.9%) | 2.1% (0.2-3.9%) |
| Manufacturing (n=70) | **41.9% (29.5-54.4%)** | 67.8% (54.3-81.2%) | 2.79 (2.25-3.33) | **70.8% (55.9-85.7%)** | **19.3% (6.8-31.8%)** | 1 | 0 (omitted) |
| Retail (n=240) | 29.1% (20.9-37.2%) | 59.2% (52.0-66.3%) | 3.35 (2.81-3.90) | **66.7% (58.0-75.3%)** | 13.0% (8.2-17.8%) | 72.5% (52.5-92.5%) | 3.6% (0.4-6.7%) |
| Hotels (n=181) | **32.6% (25.1-40.1%)** | 62.0% (53.4-70.5%) | 2.86 (2.42-3.31) | **63.5% (54.0-72.9%)** | 6.0% (2.6-9.4%) | **70%** | 1.8% (0-3.5%) |
| Financial (n=81) | 17.2% (9.0-25.5%) | 67.0% (56.9-77.0%) | 4.31 (3.32-5.31) | 43.4% (31.7-55.1%) | 3.1% (0-6.7%) | 41.8% (0-85.6%) | 1.8% (0-4.6%) |
| Public administration (n=145) | **33.6% (23.4-43.7%)** | 63.2% (53.4-73.1%) | 3.30 (2.65-3.95) | **57.1% (44.7-69.4%)** | 13.2% (6.9-19.5%) | 96.9% (90.4-100%) | 0.4% (0-1.2%) |
| Education (n=397) | 23.2% (17.6-28.9%) | 59.4% (53.0-65.8%) | 3.58 (2.92-4.24) | 38.7% (31.7-45.7%) | 9.3% (5.7-13.0%) | 76.2% (62.0-90.4%) | 2.2% (0.5-3.9%) |
| Health/social work (n=153) | 25.7% (15.7-35.7%) | 54.1% (44.6-63.6%) | 2.77 (2.18-3.35) | 47.3% (36.8-57.8%) | 7.8% (2.3-13.4%) | 83.4% (57.0-100%) | 1.3% (0-3.3%) |
| Community services (n=69) | 26.3% (13.0-39.7%) | 58.9% (44.2%-73.7%) | 3.64 (2.79-4.48) | 47.7% (31.4-64.0%) | **15.1% (5.7-24.5%)** | 85.8% (66.0-100%) | 2.2% (0-5.4%) |
| Domestic work (n=679) | **27.6% (23.2-32.0%)** | 46.5% (41.7-51.3%) | 3.18 (2.75-3.62) | **57.1% (51.6-62.7%)** | **19.8% (15.8-23.8%)** | 77.4% (68.3-86.4%) | 4.5% (2.6-6.3%) |

**Table SDC3b. Lifetime migration characteristics and HIV-related outcomes by occupation in men aged 15-64 years**

|  | **Proportion away from home for more than a month in the past 3 years % (95% CI)** | **Proportion who have lived in another region**  **% (95% CI)** | **Lifetime number of sex partners- mean (95% CI)** | **Proportion with a non-marital**  **partner in past year % (95% CI)** | **Proportion HIV-seropositive % (95% CI)** | **Proportion of PLHIV with VLS**  **% (95% CI)** | **Proportion of all participants with viremia**  **% (95% CI)** |
| --- | --- | --- | --- | --- | --- | --- | --- |
| **Occupation** |  |  |  |  |  |  |  |
| No occupation (n=3,589) | 25.5% (23.3-27.7%) | 46.1% (43.7-48.5%) | 5.3 (4.8-5.9) | **68.7% (66.2-71.2%)** | 10.3% (9.2-11.5%) | 74.8% (69.1-80.4%) | 2.6% (1.9-3.3%) |
| Agriculture (n=782) | 33.5% (28.9-38.2%) | 55.9% (51.3-60.5%) | 7.2 (6.0-8.5) | 41.2% (35.3-47.1%) | 7.6% (5.1-10.1%) | 54.9% (39.8-69.9%) | 3.4% (1.7-5.1%) |
| Fishing (n=63) | 53.5% (39.8-67.1%) | **92.0% (86.9-97.1%)** | **9.4 (5.6-13.1)** | **69.6% (59.6-79.5%)** | **13.8% (6.0-21.7%)** | **49.6% (21.7-77.4%)** | **7.0% (1.7-12.2%)** |
| Mining (n=111) | 30.7% (19.4-42.0%) | 73.8% (62.4-85.1%) | 11.1 (8.6-13.6) | 47.7% (34.6-60.7%) | 4.1% (1.2-6.9%) | 65.3% (40.6-90.0%) | 1.4% (0.1-2.7%) |
| Manufacturing (n=126) | 38.9% (29.2-48.6%) | 77.9% (70.7-85.0%) | 8.0 (5.3-10.8) | 48.9% (36.6-61.3%) | 8.1% (1.6-14.6%) | 60.2% (2.5-100%) | 3.2% (0-8.2%) |
| Construction (n=551) | 49.4% (44.8-54.0%) | 73.3% (69.0-77.5%) | 10.0 (8.1-12.0) | **63.9% (58.5-69.3%)** | 8.8% (6.2-11.3%) | 65.9% (52.7-79.1%) | 3.0% (1.6-4.4%) |
| Retail (n=218) | 37.4% (30.1-44.8%) | 69.2% (62.1-76.3%) | 9.2 (6.4-12.0) | 55.6% (47.3-63.9%) | 7.6% (4.0-11.2%) | 74.0% (52.6-95.5%) | 2.0% (0.2-3.7%) |
| Hotels (n=57) | 47.5% (29.2-65.8%) | 78.7% (65.0-92.4%) | 8.0 (5.1-10.8) | **66.8% (49.7-84.0%)** | 11.9% (4.6-19.3%) | 83.9% (62.6-100%) | 1.9% (0-4.6%) |
| Transport (n=138) | 33.8% (24.0-43.5%) | 77.4% (67.9-86.8%) | 13.1 (9.3-16.9) | 51.4% (40.3-62.5%) | 12.9% (6.2-19.5%) | 57.8% (31.9-83.8%) | **5.4% (1.4-9.5%)** |
| Public administration (n=171) | 43.9% (34.9-53.0%) | 77.4% (69.3-85.5%) | 16.3 (10.8-21.8) | 46.0% (37.1-54.9%) | 9.6% (4.5-14.8%) | 78.4% (56.6-100%) | 2.1% (0-4.3%) |
| Education (n=155) | 27.2% (18.1-36.3%) | 66.0% (56.0-76.1%) | 10.2 (6.2-14.3) | 51.8% (43.1-60.5%) | 3.1% (0.7-5.4%) | 1 | 0 (omitted) |
| Health/social work (n=61) | 27.0% (12.3-41.8%) | 71.0% (58.5-83.6%) | 10.4 (6.7-14.1) | 50.5% (34.8-66.2%) | 12.0% (1.5-22.6%) | 39.2% (0-89.2%) | 7.3% (0-17.1%) |
| Community services (n=67) | 34.4% (19.3-49.5%) | 67.8% (53.8-81.8%) | 16.8 (5.6-28.0) | 48.5% (28.7-68.3%) | 10.7% (3.7-17.7%) | 43.9% (11.7-76.0%) | 6.0% (0-12.1%) |
| Domestic work (n=381) | 28.8% (22.9-34.8%) | 45.5% (38.7-52.3%) | 7.0 (5.4-8.6) | 56.5% (49.1-64.0%) | 11.1% (7.0-15.3%) | 60.0% (41.8-78.3%) | 4.5% (1.8-7.1%) |

**Supplemental Digital Content 4. Results of the regional analysis of HIV indicators and migration**

NAMPHIA and other studies have demonstrated that there is substantial regional variation in HIV prevalence, and treatment coverage [[3](#_ENREF_3),[13](#_ENREF_13),[14](#_ENREF_14)]. A previous study of mobility in Namibia using cell phone data paired with the 2013 Demographic and Health Survey found that movement occurred in networks, often within defined geographic areas, and that these corresponded to sexual transmission communities [[3](#_ENREF_3),[15](#_ENREF_15),[16](#_ENREF_16)]. Here we describe regional variation in HIV and viral load suppression as well as mobility patterns.

**Table SDC4. Regional distribution of adults aged 15-64 years by current and previous residence, Namphia 2017**

| **Region** | **Current residence**  **N (%) (n=16,939)** | **Proportion of in-migrants who are recent**  **% (95% CI)** | **Proportion of total who have lived outside Namibia**  **% (95% CI)** | **HIV prevalence**  **% (n=16,939)** | **Prevalence of VLS in PLHIV % (n=2,446)** | **Previous residence**  **N (%)***  **(n=8,778)** |
| --- | --- | --- | --- | --- | --- | --- |
| **Coastal** | | | | | | |
| Kunene | 908 (3.6) | **19.0 (13.6-24.4)** | 2.6 (0.9-4.3) | 7.6 (4.9-10.3) | 55.2 (40.6-69.7) | 341 (3.1) |
| !Karas | 829 (3.9) | 14.9 (9.6-20.2) | 3.2 (0.1-6.3) | 9.7 (7.3-11.9) | 68.6 (58.9-78.3) | 627 (5.3) |
| Erongo | 656 (8.3) | **18.8 (13.2-24.5)** | 7.4 (0.2-14.7) | 10.6 (5.2-16.0) | 75.0 (59.9-90.1) | **1181 (10.0)** |
| Hardap | 1076 (3.8) | 14.5 (9.4-19.5) | 2.5 (1.3-3.7) | 9.3 (5.5-13.1) | 60.6 (52.0-69.2) | 418 (3.9) |
| **Northern** | | | | | | |
| Zambezi | 483 (4.0) | 16.3 (11.1-21.5) | **19.8 (13.2-26.4)** | **22.3 (17.5-27.0)** | 77.1 (70.8-83.4) | 278 (2.4) |
| Kavango East | 1406 (5.8) | 15.7 (12.8-18.6) | 3.1 (2.5-3.7) | **14.5 (11.5-17.4)** | 72.3 (63.9-80.6) | 620 (4.7) |
| Kavango West | 1009 (3.3) | 6.6 (3.9-9.3) | 1.4 (0.5-2.3) | **12.1 (9.3-14.9)** | 78.7 (68.2-89.2) | 415 (3.0) |
| Oshikoto | 1219 (6.9) | 11.6 (8.3-14.8) | 3.5 (2.2-4.8) | **17.3 (13.9-20.7)** | 79.7 (75.2-84.3) | 973 (9.3) |
| Ohangwena | 1995 (9.3) | 10.5 (8.7-12.2) | 4.3 (3.1-5.4) | 17.9 (16.0-19.8) | 86.2 (81.9-90.4) | **1115 (11.9)** |
| Omusati | 1923 (9.1) | 11.5 (8.9-14.2) | 5.1 (3.8-6.4) | **16.9 (14.9-18.9)** | 83.0 (77.7-88.3) | **1126 (12.1)** |
| Oshana | 1341 (8.8) | **21.1 (17.6-24.6)** | 4.3 (3.1-5.6) | **15.8 (11.9-19.5)** | 84.9 (78.9-90.9) | 930 (8.9) |
| **Central** | | | | | | |
| **Omaheke** | 1240 (2.7) | **23.3 (19.1-27.4)** | 4.8 (2.5-7.2) | 8.4 (6.7-10.2) | 56.3 (48.7-63.8) | 346 (3.4) |
| Otjozondjupa | 1126 (7.4) | **18.2 (15.2-21.1)** | 3.5 (1.4-5.6) | 8.5 (5.9-10.9) | 72.8 (67.1-78.5) | 803 (6.3) |
| Khomas | 1728 (23.1) | 14.8 (12.6-17.0) | 9.9 (8.2-11.6) | 8.3 (6.6-10.1) | 73.6 (61.8-85.3) | **2120 (15.7)** |

**Supplemental Digital Content 5. Figure. Joint United Nations Programme on AIDS/HIV (UNAIDS) 90-90-90 indicators by migration status in Namibia, 2017**


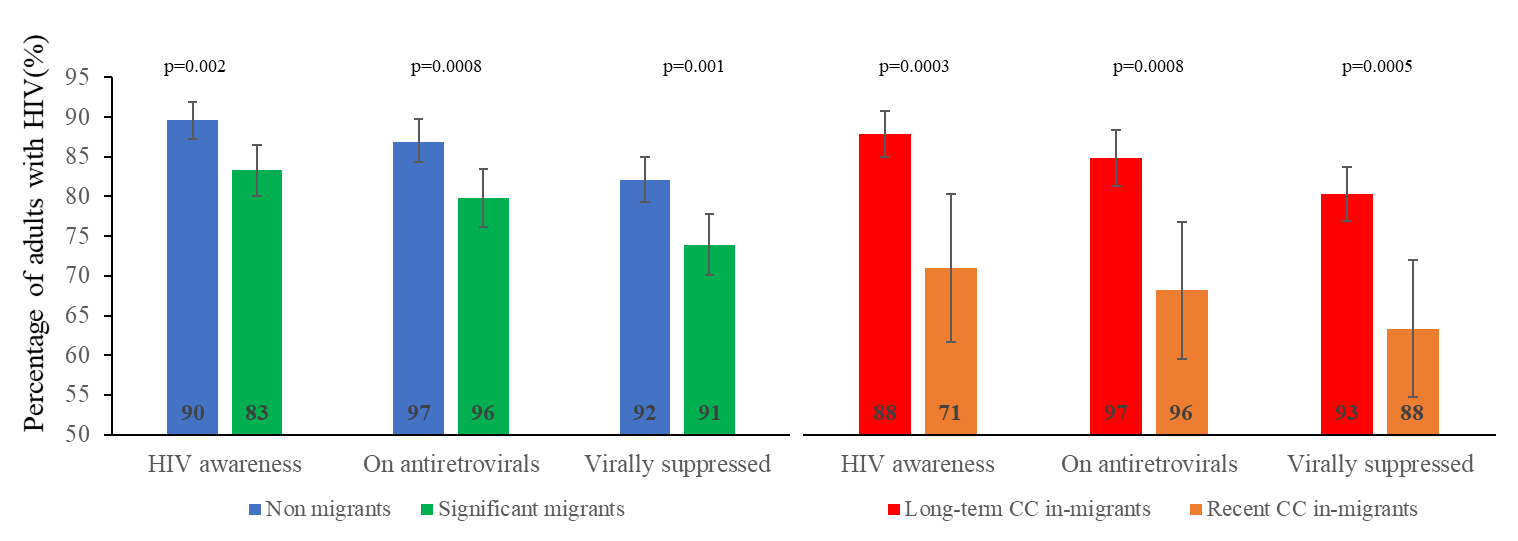


The UNAIDS 90-90-90 targets are 90% of people living with HIV (PLHIV) are aware of their status; of these, 90% are receiving antiretroviral therapy; and of these, 90% are virally suppressed. Inset numbers are conditional percentages, and bar height indicates the proportion of all PLHIV. Antiretroviral status was determined by self-report and/or those who tested positive for nevirapine, lopinavir, or efavirenz. P-values were calculated using chi-squared analysis on weighted data and indicate significance of difference between overall proportions. There were no significant differences between conditional percentages. Derivation of the classification of migrants is described in SDC 1 (CC=cross-community).

**Supplemental Digital Content- References**

1. Namibia Statistics Agency. Namibia Population and Housing Census 2011. 2011.

2. Westat. Namibia (NAMPHIA) technical report. 2018 Accessed May 1 2019.

3. The Namibia Ministry of Health and Social Services and ICF International. The Namibia Demographic and Health Survey 2013. Windhoek, Namibia2014.

4. Mmbaga EJ, Leyna GH, Hussain A, Mnyika KS, Sam NE, Klepp KI. The role of in-migrants in the increasing rural HIV-1 epidemic: results from a village population survey in the Kilimanjaro region of Tanzania. Int J Infect Dis. 2008;12(5):519-25.

5. Olawore O, Tobian AAR, Kagaayi J, Bazaale JM, Nantume B, Kigozi G, et al. Migration and risk of HIV acquisition in Rakai, Uganda: a population-based cohort study. Lancet HIV. 2018;5(4):e181-e9.

6. Chang LW, Grabowski MK, Ssekubugu R, Nalugoda F, Kigozi G, Nantume B, et al. Heterogeneity of the HIV epidemic in agrarian, trading, and fishing communities in Rakai, Uganda: an observational epidemiological study. Lancet HIV. 2016;3(8):e388-e96.

7. Kate Grabowski M, Lessler J, Bazaale J, Nabukalu D, Nankinga J, Nantume B, et al. Migration, hotspots, and dispersal of HIV infection in Rakai, Uganda. Nat Commun. 2020;11(1):976.

8. Palk L, Blower S. Brief report: Mobility and circular migration in Lesotho: implications for transmission, treatment, and control of a severe HIV epidemic. J Acquir Immune Defic Syndr. 2015;68(5):604-8.

9. Faturiyele I, Karletsos D, Ntene-Sealiete K, Musekiwa A, Khabo M, Mariti M, et al. Access to HIV care and treatment for migrants between Lesotho and South Africa: a mixed methods study. BMC Public Health. 2018;18(1):668.

10. Bygrave H, Kranzer K, Hilderbrand K, Whittall J, Jouquet G, Goemaere E, et al. Trends in loss to follow-up among migrant workers on antiretroviral therapy in a community cohort in Lesotho. PLoS One. 2010;5(10):e13198.

11. Dobra A, Barnighausen T, Vandormael A, Tanser F. Space-time migration patterns and risk of HIV acquisition in rural South Africa. AIDS. 2017;31(1):137-45.

12. Tansey E, Theyise N, Borland R, West H. Southern Africa ports as spaces of HIV vulnerability: case studies from South Africa and Namibia. Int Marit Health. 2010;62(4):233-40.

13. Ministry of Health and Social Services (MOHSS) Namibia. Namibia Population-based HIV Impact Assessment (NAMPHIA) 2017: Final Report. Windhoek, Namibia2019.

14. HIV in Namibia. SAfAIDS News. 1997;5(3):8.

15. Valdano E, Okano J, Colizza V, Blower S, editors. Mobility patterns create dynamic widely dispersed risk networks in Namibia. Conference on retroviruses and opportunistic infections; 2019 March 4-7, 2019; Seattle, Washington. Seattle, Washington2019.

16. Okano JT, Sharp K, Valdano E, Palk L, Blower S. HIV transmission and source-sink dynamics in sub-Saharan Africa. Lancet HIV. 2020;7(3):e209-e14.
